# Halofantrine Hydrochloride Acts as an Antioxidant Ability Inhibitor That Enhances Oxidative Stress Damage to *Candida albicans*

**DOI:** 10.3390/antiox13020223

**Published:** 2024-02-09

**Authors:** Juan Xiong, Li Wang, Zhe Feng, Sijin Hang, Jinhua Yu, Yanru Feng, Hui Lu, Yuanying Jiang

**Affiliations:** Department of Pharmacy, Shanghai Tenth People’s Hospital, School of Medicine, Tongji University, Shanghai 200072, China

**Keywords:** halofantrine hydrochloride, oxidative response inhibitor, oxidative damage agents, *Candida albicans*

## Abstract

*Candida albicans*, a prominent opportunistic pathogenic fungus in the human population, possesses the capacity to induce life-threatening invasive candidiasis in individuals with compromised immune systems despite the existence of antifungal medications. When faced with macrophages or neutrophils, *C. albicans* demonstrates its capability to endure oxidative stress through the utilization of antioxidant enzymes. Therefore, the enhancement of oxidative stress in innate immune cells against *C. albicans* presents a promising therapeutic approach for the treatment of invasive candidiasis. In this study, we conducted a comprehensive analysis of a library of drugs approved by the Food and Drug Administration (FDA). We discovered that halofantrine hydrochloride (HAL) can augment the antifungal properties of oxidative damage agents (plumbagin, menadione, and H_2_O_2_) by suppressing the response of *C. albicans* to reactive oxygen species (ROS). Furthermore, our investigation revealed that the inhibitory mechanism of HAL on the oxidative response is dependent on Cap1. In addition, the antifungal activity of HAL has been observed in the *Galleria mellonella* infection model. These findings provide evidence that targeting the oxidative stress response of *C. albicans* and augmenting the fungicidal capacity of oxidative damage agents hold promise as effective antifungal strategies.

## 1. Introduction

*Candida albicans*, a significant opportunistic pathogenic fungus in humans, has the potential to cause fatal invasive candidiasis in immunocompromised patients [1,2], despite the availability of antifungal drugs such as polyenes, azoles, and echinocandins [3]. The primary defense mechanism against *C. albicans* involves phagocytosis mediated by innate immune cells, including macrophages and neutrophils [4]. Upon engulfment of *C. albicans*, innate immune cells employ a major antifungal strategy by generating toxic reactive oxygen species (ROS), resulting in oxidative damage to *C. albicans* [5,6]. Indeed, ROS have the capability to initiate the oxidation of proteins, lipids, and nucleic acids, leading to impaired functionality of these vital biological macromolecules, ultimately triggering programmed cell death in the fungal pathogen [7]. *C. albicans* exhibits the ability to withstand oxidative stress by means of antioxidant enzymes, namely catalase, glutathione peroxidase, and superoxide dismutase when confronted with macrophages or neutrophils [5]. In individuals with profound innate immunodeficiency, such as those suffering from cancer, organ transplantation, and acquired immune deficiency syndrome (AIDS), *C. albicans* can persist in the bloodstream and establish colonies within internal organs, leading to the development of invasive candidiasis [8]. Thus, augmenting oxidative stress in innate immune cells against *C. albicans* represents a potential therapeutic strategy for managing invasive candidiasis.

Augmenting intracellular ROS levels in *C. albicans* represents a viable fungicidal approach akin to using amphotericin B and miconazole, which results in cell death [9,10,11]. Our prior investigations have demonstrated that increasing ROS levels in *C. albicans* using baicalein, berberine, and osthole enhances the antifungal efficacy of antifungal agents [12,13,14]. However, the generation of ROS, which is cytotoxic to *C. albicans*, is contingent upon dysfunctions in the tricarboxylic acid (TCA) cycle and the respiratory chain and may also prove toxic to mammalian cells. An alternative antifungal strategy involves weakening *C. albicans*’ resistance to oxidative damage. Cationic stress, resulting from an increased influx of K^+^ into the phagosome of innate immune cells [15], has been shown to inhibit *C. albicans*’ response to oxidative stress and render it highly susceptible [16,17]. The regulation of *C. albicans*’ response to ROS primarily depends on the transcription factors Cap1 and Hog1 [5]. The deletion of *CAP1* and *HOG1* genes in *C. albicans* rendered the organism susceptible to oxidative stress [18] and reduced its resistance to host cell-mediated killing, leading to diminished virulence in *Caenorhabditis elegans*, *Galleria mellonella*, and mouse infection models [17,19,20]. Consequently, targeting the response of *C. albicans* to ROS and augmenting the fungicidal efficacy of innate immune cells represents a promising therapeutic approach. Nevertheless, the number of compounds that function as antifungal agents through this mechanism remains limited.

Here, we performed a high throughput screening of a Food and Drug Administration (FDA)-approved drug library and identified that halofantrine hydrochloride (HAL) could enhance the antifungal activities of oxidative damage agents (plumbagin, menadione, and H_2_O_2_) by suppressing *C. albicans’* response to ROS. We further found that the mechanism of inhibiting the oxidative response action of HAL depends on Cap1. In addition, the antifungal activity of HAL has been observed in the *G. mellonella* infection model. These findings demonstrate that inhibiting *C. albicans’* response to oxidative stress and enhancing the fungicidal ability of innate immunity cells is a promising antifungal strategy.

## 2. Materials and Methods

### 2.1. Strains, Primers, Agents, and Cultural Conditions

The present study utilized *C. albicans’* strains, which are documented in Table 1, and primers, which are documented in Table 2. *C. albicans’* strains were cultured in a YPD medium (1% yeast extract, 2% peptone, and 2% dextrose) at 30 °C. Deletion and green fluorescence protein (GFP)-tagged mutants were constructed using a synthetic medium (0.67% yeast nitrogen base without amino acids, 2% dextrose, 2% agar). The FDA-approved drug library (MedChemExpress, Shanghai, China) consisted of 2372 drugs that were dissolved in dimethylsulfoxide (DMSO) at 10 mM. Plumbagin (Topscience, Shanghai, China) and menadione (Topscience, Shanghai, China) were dissolved in DMSO to prepare the stock solutions at 6.4 mg/mL and 102.4 mM, respectively.

### 2.2. High-Throughput Screening

To identify drugs that potentiate oxidative stress damage to *C. albicans*, a logarithmic long-term culture of *C. albicans* was diluted to a concentration of 1 × 10^3^ cells/mL in a YPD medium containing 1 μg/mL plumbagin. Subsequently, 1 μL of each FDA-approved drug was added to 199 μL of the cell suspension, resulting in a concentration of 50 μM in each well of a 96-well plate, which was then incubated at 30 °C for 48 h. The criterion for drug inclusion was a growth reduction of >50%, as determined by OD_600_ values, in a combination of an FDA-approved drug and plumbagin compared to plumbagin monotherapy.

### 2.3. Minimum Inhibition Concentration (MIC) Assay

The MIC assay was conducted in accordance with the guidelines outlined in the Clinical and Laboratory Standards Institute (CLSI) M27-A3. YPD medium was utilized, with strains inoculated at a concentration of approximately 1 × 10^3^ cells/mL and 100 μL per well in 96-well plates. Serial dilutions of compounds ranging from 50 μM to 0.1 μM were added to each well. These plates were then incubated at 30 °C for 24 h. Following incubation, the optical densities were measured at an absorbance of 600 nm, using a Multiskan Sky (Thermo Fisher Scientific, Waltham, MA, USA). The MIC was determined as the concentration of the compound that suppressed 50% or more of cellular growth, as evidenced by the OD_600_ measurement, in comparison to the control.

### 2.4. Dose–Matrix Titration Assay

The dose–matrix titration assay, as outlined in the reference, was employed to evaluate the synergistic effects of drugs [22]. In summary, drug A was diluted in a two-fold serial manner across columns of a 96-well plate, with each well containing 50 μL of drug A at a concentration four times higher than the final drug concentration. Similarly, drug B was dispensed in a two-fold serial dilution manner across rows of the plate, with each well containing 50 μL of drug B at a concentration four times higher than the final drug concentration. Following this, a volume of 50 μL of the diluted drug B was subsequently transferred to the plate containing drug A. Subsequently, a volume of 100 μL of overnight diluted *C. albicans* cultures (1 × 10^3^ cells/mL) was added to all wells containing the drugs and incubated at 30 °C for 24 h. The synergy between drugs A and B was assessed using the Loewe additivity model, employing the fractional inhibitory concentration index (FICI). Synergism is indicated by an FICI value of ≤0.5, additive effects by an FICI value of 0.5 < FICI ≤ 1, indifference by an FICI value of 1 < FICI ≤ 4, and antagonism by an FICI value greater than 4 [23].

### 2.5. Disruption of Target Genes

Fusion PCR methodology was utilized to generate null mutants of genes of interest from the *C. albicans*’ strain SN152, as previously described in reference [21]. In brief, the flanking sequences of the target genes were amplified using primers P1 and P3 or P4 and P6, with the genomic DNA of SN152 serving as the template. The selectable *HIS1* or *ARG4* marker was then amplified using universal primers 2 and 5, with plasmids pSN52 or pSN69 as the template. Subsequently, a fusion product was generated using primers P1 and P6, with three PCR products serving as the template. The transformation was selected on synthetic media supplemented with the necessary auxotrophic supplements.

### 2.6. Quantitative Real-Time PCR (qRT-PCR) Analysis

The methodology for total RNA extraction and qRT-PCR was conducted according to the procedures described in a previous study [24]. To ensure the absence of genomic DNA contamination, the isolated RNA was subjected to DNase I treatment (Takara, Beijing, China). First-strand cDNAs were synthesized using a reverse transcription PCR cDNA synthesis kit (Takara, China). Triplicate independent qRT-PCR analyses were performed using the Roche Lightcycler 96 Fluorescence Quantitative PCR Instrument and TB Green Premix Ex TaqTM II (Takara, China). The *ACT1* gene served as the internal control. 

### 2.7. C-Terminal of Proteins Tagging GFP

A PCR-based approach was employed to amplify the desired DNA cassettes within plasmid pCPC64 for tagging the C-terminal of Cap1 with GFP [25]. The generation of DNA cassettes with 78 bp homology regions to the *CAP1* gene was achieved through two rounds of PCR utilizing primers F1 plus R1 and F2 plus R2, respectively. The resulting product was subsequently transformed into *C. albicans* cells to generate a mutant strain with the C-terminal of Cap1 tagged with GFP. 

### 2.8. Western Blot Analysis

*C. albicans’* cells were washed with sterile water once and then were subjected to cell lysis using the Bead Ruptor12 System (OMNI International, Kennesaw, GA, USA) in lysis buffer (PBS containing 5 mM EDTA (pH 8.0), 1 mM PMSF, 1.0% Protease Inhibitor Cocktail). Proteins were separated by 4–20% SDS–PAGE and transferred to a PVDF membrane. After blocking, anti-GFP antibodies (Santa Cruz, Dallas, TX, USA) or anti-tubulin antibodies (Abbkine, Atlanta, GA, USA) were used for probing GFP-tagged proteins or the tubulin, which were then detected using the secondary antibody goat anti-mouse IgG-HRP (Santa Cruz) and the Pierce™ ECL system (Thermo Fisher Scientific, Waltham, MA, USA) [26].

### 2.9. G. mellonella Infection Model

*G. mellonella* larvae, obtained from the Tianjin Huiyude Biotech Company, were selected based on an average weight of 300 mg and randomly assigned to four groups (*n* = 10 per group), with any larvae displaying signs of melanization being excluded. The larvae were infected with 5 μL of an SN152 suspension (7.0 × 10^5^ cells/larvae) using a Hamilton syringe and subsequently treated with a single injection of HAL (0, 0.5, 1, 2 mg/kg). All *G. mellonella* larvae were incubated at 30 °C for eight days. The mortality of *G. mellonella* was evaluated daily and subjected to statistical analysis using the Kaplan–Meier method, specifically employing the log-rank test [22,27].

## 3. Results

### 3.1. HAL Enhances the Antifungal Activities of Oxidative Damage Agents

**Figure 1:**
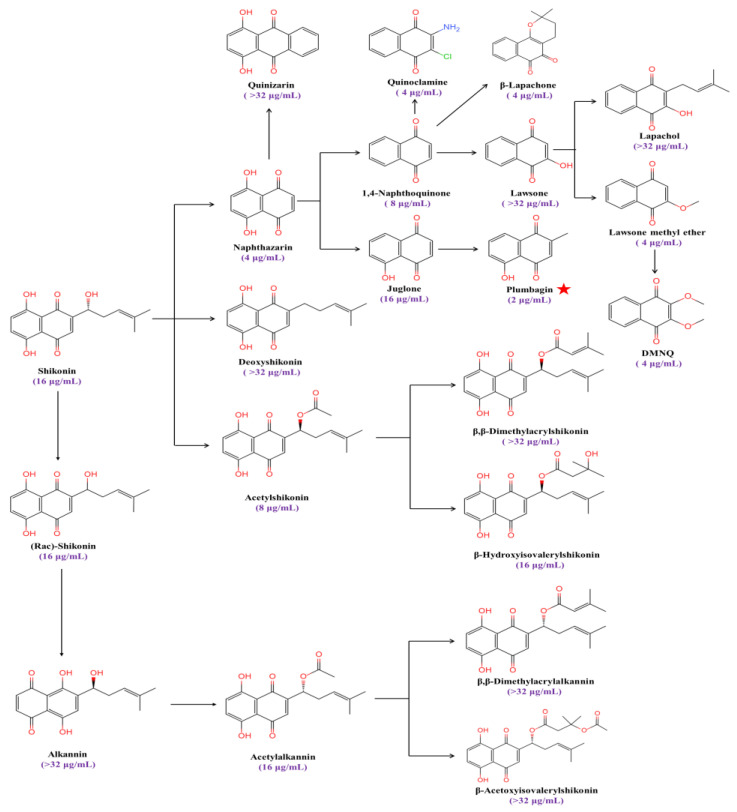
The MIC values of anthraquinone analogs against *C. albicans*. The red star symbolizes the compound exhibiting the lowest MIC value against *C. albicans*.

Plumbagin (5-hydroxy-2-methyl-1,4-naphthoquinone) is a strong inducer of ROS and causes oxidative stress in fungi [28,29]. We found that the MIC value of plumbagin against *C. albicans’* SC5314 is 2 μg/mL, with the strongest antifungal activity among a series of anthraquinone analogs (Figure 1).

**Figure 2:**
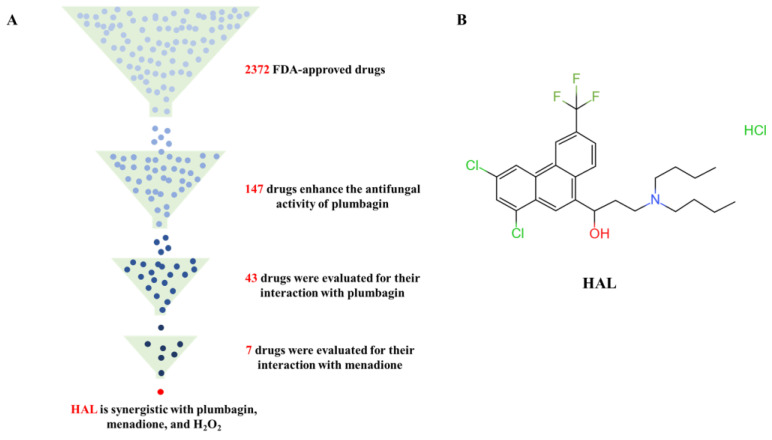
(**A**) Identification of HAL for enhancing the antifungal activity of ROS inducers from an FDA-approved compound library. (**B**) HAL chemical structure.

In this study, we performed a high-throughput screening of the FDA-approved drug library containing 2372 drugs to determine candidate agents that are synergistic with the ROS inducer plumbagin. Previous studies demonstrated that loss of Cap1 and Hog1 increased the susceptibility of *C. albicans* to oxidizing agents [10,30,31] but had no function in the growth of *C. albicans* [32]. Therefore, we utilize three criteria to screen candidate inhibitors of the oxidative stress response of *C. albicans*: a candidate inhibitor should (1) have weak antifungal activity but (2) enhance the antifungal activity of ROS inducers, and (3) ROS inducers can also enhance its antifungal activity. We tested the antifungal activity of 50 μM of each of the 2372 drugs on *C. albicans’* SN152, grown in YPD medium with 1 μg/mL plumbagin in 96-well plates. We found that 147 compounds enhanced the antifungal activity of plumbagin, and the relative growth of *C. albicans* treated with the combination of candidate compounds and plumbagin is less than 50% of that of plumbagin alone after incubation at 30 °C for 24 h (Figure 2A, Table 3). We further investigated the ability of these compounds to enhance the antifungal activity of plumbagin (1 μg/mL), using the MIC assay by diluting these candidate compounds from 50 μM to 0.1 μM in a 2-fold ratio. We excluded 30 drugs for subsequent study as these drugs have potent antifungal activities (MIC values are less than 0.1 μM). We precluded 10 drugs for which plumbagin antagonized their antifungal activity, and 64 for which plumbagin slightly enhanced their antifungal activities (the ratio of MIC alone to the MIC in the presence of plumbagin is equal or less than 2) (Figure 2A, Table 3). Subsequently, we used a dose–matrix titration assay to determine the interaction between the remaining 43 drugs and plumbagin by the FICI. We included seven drugs as candidate oxidative response inhibitors as they synergize with plumbagin. Among them, the combination of HAL and plumbagin had the lowest FICI value (FICI = 0.0938) (Figure 2B, Table 4). We further tested the interaction of each of the seven drugs and menadione (2-methyl-1,4-naphthoquinone), another ROS inducer [29]. Only HAL was synergistic with menadione, with an FICI value of 0.1563 (Figure 2A, Table 5). We then gauged the combination of HAL and H_2_O_2_; as expected, they are synergistic (FICI = 0.2526) (Table 6). These findings suggest that HAL is a promising synergistic agent for ROS inducers.

### 3.2. HAL Inhibits the Oxidative Stress Response in C. albicans

**Figure 3:**
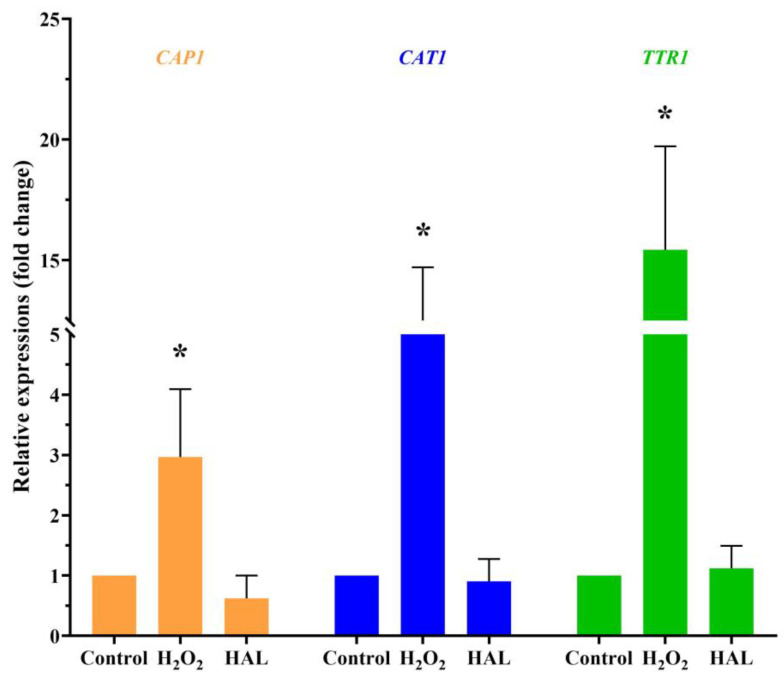
The transcription levels of the *CAP1*, *CAT1*, and *TTR1* genes in response to 10 mM H_2_O_2_ and 20 μM HAL for 1 h were measured by qRT-PCR. The significance of differences was determined by one-way ANOVA analysis, followed by Dunnett’s multiple comparisons test (* *p* < 0.05).

Is it possible that HAL acts as an oxidant that enhances the antifungal activity of ROS inducers? Exposure to cationic stress, such as sodium chloride (NaCl), inhibits the oxidative stress response of *C. albicans* [16]. If HAL is indeed an oxidant, it would be expected that HAL and NaCl would mutually enhance their respective antifungal activities. However, the interaction between HAL and NaCl appears indifferent (Table 7). On the other hand, in the presence of plumbagin, HAL and NaCl exhibit additive interactions (Table 7), indicating that HAL, like NaCl, can inhibit *C. albicans’* oxidative stress response. One of the mechanisms employed by *C. albicans* to withstand oxidative stress involves active transcriptional responses to oxidative stress. This includes the activation of specific genes, such as the *CAP1* gene (C3_02220W_A), encoding an AP-1 bZIP transcription factor, the *CAT1* gene (C1_06810W_A), encoding catalase, and the *TTR1* gene (C1_00490C_A), encoding glutaredoxin [5]. When *C. albicans* are exposed to a 10 mM concentration of H_2_O_2_ for 1 h, an upregulation in the expression levels of the three genes is observed. Conversely, a high concentration of HAL (20 μM) has minimal impact on the expression of these three genes (Figure 3). These findings indicate that HAL acts as an inhibitor of the oxidative stress response in *C. albicans*, thereby enhancing the antifungal activities of oxidative damage agents.

### 3.3. The Inhibitory Effect of HAL on Oxidative Stress Response Depends on the Cap1–Ybp1 Signaling Pathway

Three signaling pathways play a crucial role in response to ROS in *C. albicans*, including the transcriptional factor Cap1, the stress-activated protein kinase Hog1, and the DNA damage checkpoint kinase Rad53 [5]. Since HAL can inhibit the response to oxidative stress in *C. albicans*, HAL may target one of the signaling pathways that mediate *C. albicans’* responses to ROS. To verify this conjecture, we constructed the *CAP1* (C3_02220W_A), *HOG1* (C2_03330C_A), and *RAD53* (C3_03810W_A) genes’ null mutants (*cap1*Δ/*cap1*Δ, *hog1*Δ/*hog1*Δ, and *rad53*Δ/*rad53*Δ) in *C. albicans*. We found that the synergistic effect between HAL and H_2_O_2_ became indifferent in the *cap1*Δ/*cap1*Δ mutant, while their interaction remained synergistic in the *hog1*Δ/*hog1*Δ and *rad53*Δ/*rad53*Δ mutants (Table 6), suggesting that the inhibitory effect of HAL on oxidative stress response depends on the transcriptional factor Cap1.

Ybp1 is encoded by the *YBP1* (C1_13960W_A) gene in *C. albicans*, binds to cytoplasmic pools of Cap1, and forms a complex with Cap1 [19], protecting Cap1 from ubiquitin-mediated degradation [33]. Like in the *cap1*Δ/*cap1*Δ mutant, HAL can no longer enhance the antifungal activity of H_2_O_2_ against the *ybp1*Δ/*ybp1*Δ mutant (Table 6). These findings indicate that the inhibitory effect of HAL on the antioxidant ability of *C. albicans* depends on the presence of Cap1 and Ybp1.

**Figure 4:**
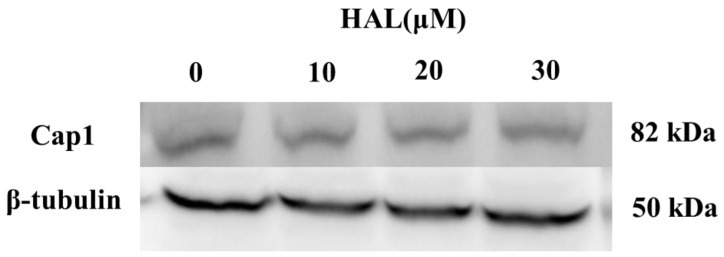
The expression levels of Cap1 in the presence (10 μM, 20 μM, and 30 μM) or absence of HAL were detected by immunoblotting.

Cap1 will degrade when it cannot form a complex with Ybp1 [19]. Therefore, we conjecture that HAL inhibits the antioxidant ability of *C. albicans* by disrupting the interaction between Cap1 and Ybp1 and improving the degradation of Cap1. To verify this hypothesis, we tagged the C-termini of Cap1 with a GFP tag [25]. We then cultured the Cap1–GFP mutant in the presence (10 μM, 20 μM, 30 μM) or absence of HAL, respectively, and then treated it with 10 mM H_2_O_2_ for 1 h. We tested the expression of Cap1 using an anti-GFP antibody and found that the HAL did not affect the Cap1 levels in the presence of H_2_O_2_ (Figure 4), which refuted our conjecture that HAL should disrupt the interaction of Cap1 and Ybp1.

Following exposure of *C. albicans* to H_2_O_2_, Cap1 is activated by oxidation through a glutathione peroxidase Gpx3 [19]. Oxidized Cap1 (Cap1^ox^) can detach from the nuclear export factor Crm1, resulting in the accumulation of Cap1 in the nucleus and inducing the expression of genes with antioxidant functions [5]. When HAL and H_2_O_2_ are used together, they exhibit additive interactions against the *gpx3*Δ/*gpx3*Δ mutant (Table 6), suggesting that, like Gpx3, HAL may inhibit the activation and nucleation of Cap1 by inhibiting its oxidation or phosphorylation, thereby inhibiting the antioxidant activity of Cap1.

### 3.4. HAL Exhibits Antifungal Activity in the G. mellonella Infection Model

**Figure 5:**
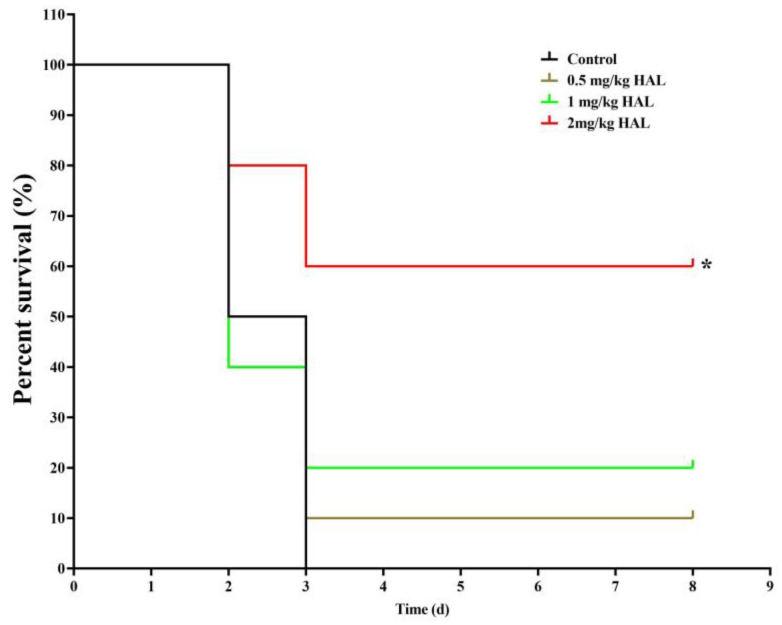
HAL exhibits antifungal efficacy in the *G. mellonella* infection model. Survival curves of larvae infected with SN152 (7.0 × 10^5^ cells/larvae) and injected with 0 mg/kg, 0.5 mg/kg, 1 mg/kg, 2 mg/kg doses of HAL. Each curve represents a group of 10 larvae (*n* = 10), monitored daily for survival for up to 8 days after infection. The significance of differences was determined by the Kaplan–Meier method, followed by the log-rank test (* *p* < 0.05).

We used the *G. mellonella* infection model to test the antifungal efficacy of HAL in vivo, as *G. mellonella* utilizes phagocytic cells (hemocytes) as part of their host defense [34,35]. We divided the *G. mellonella* larvae into four groups, with ten larvae in each group: (1) a control group receiving no drug treatment, (2) a group treated with 0.5 mg/kg HAL, (3) a group treated with 1 mg/kg HAL, and (4) a group treated with 2 mg/kg HAL. Our observations revealed a mortality rate of 100% in the control group throughout the 8-day observation period. However, upon administration of 2 mg/kg HAL, the mortality rate of infected mice decreased to 40% (Figure 5). These in vivo experiments prove that HAL has antifungal activity against *C. albicans* in the *G. mellonella* infection model.

## 4. Discussion

In this study, we performed a high-throughput screening of an FDA-approved drug library and identified that HAL could enhance the antifungal activities of oxidative damage agents by suppressing *C. albicans’* response to ROS. We further found that HAL inhibits the oxidative stress response of *C. albicans*, depending on Cap1. In addition, the antifungal activity of HAL has been observed in the *G. mellonella* infection model. These findings demonstrated that inhibiting the oxidative stress response of *C. albicans*, thereby enhancing the antifungal activity of oxidative damage agents and innate immunity cells, is a promising antifungal strategy.

Candidiasis, primarily attributed to *C. albicans*, presents a significant risk to human health, and the availability of effective drugs for its treatment remains limited [26]. Consequently, there is an imperative to explore the development of novel antifungal medications. However, the comprehensive creation of new antifungal compounds with potent antifungal properties and optimal safety profiles, rendering them clinically valuable, necessitates substantial investments of time and resources. Considering this, repurposing FDA-approved drugs to treat candidiasis can circumvent the need for extensive safety assessments and reduce the associated time and financial burdens [36]. Numerous studies have demonstrated that FDA-approved non-antifungal drugs, such as statins and sertraline, can exhibit antifungal properties [37,38]. Following this drug repurposing strategy, our study conducted a high-throughput screening of a library comprising 2372 FDA-approved compounds and found that HAL effectively enhanced the antifungal activities of oxidative damage agents and exhibited antifungal activity in the *G. mellonella* infection model. 

Inhibiting the oxidative stress response of *C. albicans* presents a promising strategy for enhancing the efficacy of antifungal treatment. Phagocytic cells are known to exert antifungal effects by generating ROS to eliminate *C. albicans*. Therefore, it is plausible to increase the intracellular ROS levels of *C. albicans*, intensify their oxidative damage, and synergistically enhance the antifungal activity of phagocytic cells or oxidative damage agents. However, there is a significant degree of conservation in the proteins comprising the mitochondrial respiratory chain between *C. albicans* and mammals. Consequently, the dysfunction of the mitochondrial respiratory chain induced by these compounds not only enhances the generation of ROS in *C. albicans’* cells but also elevates ROS levels in mammalian cells. This ultimately leads to oxidative damage and cytotoxicity in mammalian cells, thereby restricting the potential clinical utility of these compounds. Consequently, it becomes imperative to investigate novel approaches to intensify the oxidative damage inflicted upon *C. albicans*. Loss of Cap1 increases susceptibility to menadione, H_2_O_2_, and host phagocytes [17,19,30], suggesting that the transcriptional factor Cap1 is important for the virulence of *C. albicans* [20] and is a valuable target for antifungal treatment. In the present study, we found that HAL can be repurposed as an inhibitor of the oxidative stress response of *C. albicans* to enhance the antifungal activity of oxidative damage agents in vitro. We further found that the mechanism of inhibiting the oxidative response action of HAL depends on inhibiting the transcriptional activity of *C. albicans’* Cap1. In this study, we provide a novel antifungal strategy that inhibits the transcriptional activity of Cap1 and enhances the antifungal activity of oxidative damage agents.

Several mini-host models, including *Drosophila melanogaster*, *Caenorhabditis elegans*, and *G. mellonella*, have been utilized to investigate the pathophysiology of various fungal species [39]. Of interest is the *G. mellonella* infection model, which offers advantages such as cost effectiveness, ease of use, and independence from specialized infrastructures. The small size of *G. mellonella* larvae facilitates convenient manipulation, and their ability to withstand temperatures of 37 °C adds to their suitability for experimentation. The observation of melanization of larvae, decreased mobility, and mortality allows for easy detection of experimental outcomes [40]. The immune system of *G. mellonella* consists of phagocytic cells, which play a crucial role in the host defense by neutralizing and eliminating pathogens. This organism has been widely utilized in medical mycology research to investigate the virulence of pathogens and assess the effectiveness of antifungal treatments [41]. Thus, in this study, we employed the *G. mellonella* infection model to evaluate the antifungal properties of HAL, and our findings demonstrate that HAL effectively safeguards *G. mellonella* against *C. albicans’* infection. 

As an antimalarial drug, HAL has recently been reported to enhance the antifungal activity of amphotericin B against *Cryptococcus* and *Candida* species [42], suggesting that HAL may affect the fungal oxidative stress system since amphotericin B culminates *C. albicans* in death through the production of cytotoxic ROS [9]. In this study, we further uncovered the mechanism of HAL and its actions. The toxicity of HAL is low in *G. mellonella*, suggesting a promising clinical application. However, HAL is a blocker of delayed rectifier potassium current via the inhibition of the human-ether-a-go-go-related gene (hERG) channel [43,44]. Therefore, further research should develop a series of HAL analogs without inhibiting the hERG channel.

## 5. Conclusions

In conclusion, our study has identified that HAL has the potential to enhance the antifungal activities of oxidative damage agents (plumbagin, menadione, and H_2_O_2_) by suppressing the response of *C. albicans* to ROS. Furthermore, we discovered that the mechanism behind HAL’s inhibition of the oxidative response involves the inhibition of Cap1’s transcriptional activity. Additionally, the antifungal activity of HAL has been observed in the *G. mellonella* infection model. These findings provide evidence that targeting the oxidative stress response of *C. albicans* and enhancing the fungicidal ability of innate immunity cells could serve as a promising antifungal strategy.

## Figures and Tables

**Table 1:** Strains used in this study.

Gene Accession No.	Strain	Genotype	Source or Reference
	SC5314	Wild type	[21]
	SN152	*his1*/*his1 arg4*/*arg4 leu2*/*leu2*	[21]
3636640	*cap1*Δ/*cap1*Δ	*cap1*::*HIS1*/*cap1*::*ARG4 leu2*/*leu2*	This study
3637393	*hog1*Δ/*hog1*Δ	*hog1*::*HIS1*/*hog1*::*ARG4 leu2*/*leu2*	This study
3645633	*rad53*Δ/*rad53*Δ	*rad53*::*HIS1*/*rad53*::*ARG4 leu2*/*leu2*	This study
3636187	*ybp1*Δ/*ybp1*Δ	*ybp1*::*HIS1*/*ybp1*::*ARG4 leu2*/*leu2*	This study
3644471	*gpx3*Δ/*gpx3*Δ	*gpx3*::*HIS1*/*gpx3*::*ARG4 leu2*/*leu2*	This study
3636640	CAP1-GFP	his1/his1 arg4/arg4 CAP1/cap1::CAP1-GFP-LEU2	This study

**Table 2:** Primers used in this study.

Gene Accession No.	Gene Name	Primer Name ^a^	Primer Sequence (5′ to 3′) ^b^
	Primers for genes deletion		
3636640	*CAP1*	CAP1 P1	GATTACTAATTATTCTTTGAC
		CAP1 P3	cacggcgcgcctagcagcggGAATAAGGATAGTTGAAAATG
		CAP1 P4	gtcagcggccgcatccctgcCATTAATCAAGTTAGTGGTGG
		CAP1 P6	CTAGTTGAATCAAAGAAAGCC
3637393	*HOG1*	HOG1 P1	GCCTTGCTTATGTTCACAAAC
		HOG1 P3	cacggcgcgcctagcagcggCATTTTCTTATATGCTTTATC
		HOG1 P4	gtcagcggccgcatccctgcTCTTCAAAAATACAAGCTAGC
		HOG1 P6	TCGTAAGGACGGTATTACAGC
3645633	*RAD53*	RAD53 P1	CTAGTTTTCATCTTGATCTTG
		RAD53 P3	cacggcgcgcctagcagcggTGTAGTTTGGTAAATTAAGGG
		RAD53 P4	gtcagcggccgcatccctgcATTTAGCATATATACAAGCAT
		RAD53 P6	GAACGGAGATGGCAACATGTG
3636187	*YBP1*	YBP1 P1	GCTAGTTTATCCCCTCTTATG
		YBP1 P3	cacggcgcgcctagcagcggAAATTGAAATGGCTCAATGGT
		YBP1 P4	gtcagcggccgcatccctgcTGTATATGTATGTAACTACGT
		YBP1 P6	CTTCACCATTACCATCTCATC
3644471	*GPX3*	GPX3 P1	GCTGTCAAACCATTGGAGCTC
		GPX3 P3	cacggcgcgcctagcagcggTGATGATTGTTGATAATTGTA
		GPX3 P4	gtcagcggccgcatccctgcAAATACAGTAGTATTATACAT
		GPX3 P6	CGGGCAGGTCAATGCCAAACC
		Universal primer 2	ccgctgctaggcgcgccgtgACCAGTGTGATGGATATCTGC
		Universal primer 5	gcagggatgcggccgctgacAGCTCGGATCCACTAGTAACG
	Primers for diagnosing the genes null mutants		
3636640	*CAP1*	CAP1 Ucheck	CTGGCTGGCTTATACTCTAAC
		CAP1 Dcheck	ATCTGGATCGATCTCTGCAAG
3637393	*HOG1*	HOG1 Ucheck	AGGTAGTGTTGGTGTTATCAC
		HOG1 Dcheck	GAAGCATTTGGATAAATTGGG
3645633	*RAD53*	RAD53 Ucheck	CGAAATACGATACGTTAGACG
		RAD53 Dcheck	TTGGACATTGAGCATGTTCGG
3636187	*YBP1*	YBP1 Ucheck	GGTATTTTGGTTGGGATTGGG
		YBP1 Dcheck	TGAATGTTCTTAAACTTGCCG
3644471	*GPX3*	GPX3 Ucheck	TGTGTCATGTCACGTGATAAC
		GPX3 Dcheck	CATAGCCATCAATCTCTTGGT
8048008	*HIS1*	HIS1 Left	ATTAGATACGTTGGTGGTTC
		HIS1 Right	AACACAACTGCACAATCTGG
8049225	*ARG4*	ARG4 Left	ACACAGAGATACCTTGTACT
		ARG4 Right	ACGGAGTACCACATACGATG
	Primers for GFP-tagged C-terminals of Cap1		
3636640	*CAP1*	Cap1gfp-F1	GCTGATGTGAATCAATTACTAGAGCGAAGTATAAAACATCCCCAGGTCGACTCTAGATC
		Cap1gfp-R1	GAAATACCGTAAAATAAATTAAACCCACCACTAACTTGATTCTTTCCTGCGTTATCCTG
		Cap1gfp-F2	AAAGCTAAATGTTCTGAAAAGGGAGTAGTGATAAATACTGCTGATGTGAATCAATTACTA
		Cap1gfp-R2	ATATAAATACAAAAAAATAAAGCCAAATAGATGTCAATTGAAATACCGTAAAATAAATTA
		Cap1check-F	GAAGTTGTGCCGGCACCTCC
		Cap1check-R	AGATGATGTTGATTATGGTG
		VP8	GAATAATTCTTCACCTTTAGAGATGGT
		VP19	TGCAGATATCCATCACACTGG
	Primers for qRT-PCR		
3636640	*CAP1*	CAP1-rtF	TGGGTTCATCTTCATCGT
		CAP1-rtR	TTGGGCACTGGGTTACTT
3639495	*CAT1*	CAT1-rtF	AAGAGTTGTCCACGCTAA
		CAT1-rtR	GAACCTAATTCACCACCA
3639313	*TTR1*	TTR1-rtF	ATTGCCTCCAAATCCTAT
		TTR1-rtR	TGTTGACCACCAATAAAG
3636195	*ACT1*	ACT1-rtF	TTGATTTGGCTGGTAGAG
		ACT1-rtR	ATGGCAGAAGATTGAGAA

Annotation: ^a^ Ucheck, upstream check primer for target genes; Dcheck, downstream check primer for target genes; Left, upstream check primer for the *HIS1* and *ARG4* genes; Right, downstream check primer for the *HIS1* and *ARG4* genes; rtF, forward primer for qRT-PCR; rtR, reverse primer for qRT-PCR. ^b^ Lowercase sequences correspond to exogenous, complementary sequences that were added to primers 2, 3, 4, and 5 to facilitate mutually primed synthesis during the second round of fusion PCR.

**Table 3:** Compounds enhance the antifungal activity of plumbagin.

No.	Drugs	MIC (μM)		Fold Change of MIC (MIC_alone_/MIC_combined_)
		Alone	Combination with Plumbagin	
1	Almonertinib hydrochloride	>50	0.78	64
2	Bosutinib	>50	12.5	4
3	Ceritinib dihydrochloride	50	6.25	8
4	Cetylpyridinium chloride	6.25	1.56	4
5	Cinacalcet	>50	12.5	4
6	Clomiphene citrate	25	6.25	4
7	Dacomitinib	>50	12.5	4
8	Halofantrine hydrochloride	>50	6.25	8
9	Ilaprazole	>50	6.25	8
10	Nilotinib monohydrochloride monohydrate	>50	12.5	4
11	Pimavanserin tartrate	50	12.5	4
12	Tafenoquine Succinate	25	3.13	8
13	Triflupromazine hydrochloride	>50	6.25	8
14	Vilanterol trifenatate	>50	1.56	32
15	Vortioxetine	25	6.25	4
16	Vortioxetine hydrobromide	50	12.5	4
17	Alectinib	>50	0.78	64
18	Amiodarone hydrochloride	>50	12.5	4
19	Amphotericin B	0.78	<0.1	8
20	Benzethonium chloride	12.5	3.13	4
21	Bleomycin hydrochloride	50	6.25	8
22	Ceritinib	50	6.25	8
23	Chlorhexidine	25	3.13	8
24	Clioquinol	25	6.25	4
25	Disulfiram	12.5	3.13	4
26	Domiphen bromide	25	3.13	8
27	Ebastine	25	6.25	4
28	Fingolimod	12.5	1.56	8
29	Ibudilast	>50	12.5	4
30	Magnolol	50	6.25	8
31	Menadione	50	12.5	4
32	Olmutinib	>50	6.25	8
33	Pinaverium bromide	50	3.13	16
34	Ponatinib	>50	6.25	8
35	Rolapitant	50	12.5	4
36	Sertindole	>50	12.5	4
37	Sonidegib	>50	0.78	64
38	Sultiame	50	6.25	8
39	Tegaserod maleate	50	12.5	4
40	Telotristat ethyl	12.5	3.13	4
41	Telotristat etiprate	25	3.13	8
42	Thonzonium bromide	12.5	0.78	16
43	Triclosan	12.5	1.56	8
44	Butoconazole nitrate	<0.1	NA	NA
45	Cinacalcet hydrochloride	<0.1	NA	NA
46	Clotrimazole	<0.1	NA	NA
47	Econazole nitrate	<0.1	NA	NA
48	Efinaconazole	<0.1	NA	NA
49	Everolimus	<0.1	NA	NA
50	Fenticonazole Nitrate	<0.1	NA	NA
51	Isavuconazole	<0.1	NA	NA
52	Isoconazole nitrate	<0.1	NA	NA
53	Itraconazole	<0.1	NA	NA
54	Ketoconazole	<0.1	NA	NA
55	Luliconazole	<0.1	NA	NA
56	Micafungin sodium	<0.1	NA	NA
57	Miconazole nitrate	<0.1	NA	NA
58	Neticonazole hydrochloride	<0.1	NA	NA
59	Oxiconazole nitrate	<0.1	NA	NA
60	Posaconazole	<0.1	NA	NA
61	Rapamycin	<0.1	NA	NA
62	Sertaconazole nitrate	<0.1	NA	NA
63	Sulconazole mononitrate	<0.1	NA	NA
64	Temsirolimus	<0.1	NA	NA
65	(+)-Ketoconazole	<0.1	NA	NA
66	Amorolfine hydrochloride	<0.1	NA	NA
67	Dasatinib	<0.1	NA	NA
68	Econazole	<0.1	NA	NA
69	Isavuconazonium sulfate	<0.1	NA	NA
70	Lapatinib	<0.1	NA	NA
71	Neticonazole	<0.1	NA	NA
72	Tioconazole	<0.1	NA	NA
73	Voriconazole	<0.1	NA	NA
74	Atracurium besylate	25	50	0.50
75	Broxyquinoline	1.56	3.13	0.50
76	Cetrorelix Acetate	6.25	50	0.13
77	Revefenacin	25	50	0.50
78	Adapalene	25	50	0.50
79	Bifonazole	3.13	6.25	0.50
80	Dimetridazole	12.5	25	0.50
81	Dioscin	0.2	3.13	0.06
82	Terbinafine	1.56	3.13	0.50
83	Terbinafine hydrochloride	1.56	3.13	0.50
84	10-Undecenoic acid	>50	50	1
85	10-Undecenoic acid zinc salt	>50	50	1
86	Aclacinomycin A hydrochloride	25	12.5	2
87	Atorvastatin	12.5	12.5	1
88	Aviptadil acetate	>50	>50	1
89	Bleomycin sulfate	25	12.5	2
90	Blonanserin	>50	50	1
91	Chlorprothixene	>50	25	2
92	Chlorquinaldol	3.13	1.56	2
93	Ciclopirox	50	50	1
94	Dronedarone hydrochloride	25	12.5	2
95	Fluconazole	6.25	6.25	1
96	Fluvastatin sodium	3.13	3.13	1
97	Fosravuconazole lysine ethanolate	12.5	12.5	1
98	Josamycin	>50	50	1
99	Lonafarnib	>50	>50	1
100	L-Thyroxine sodium	>50	50	1
101	Miltefosine	50	50	1
102	Mycophenolic acid	3.13	3.13	1
103	Natamycin	25	25	1
104	Nintedanib esylate	>50	50	1
105	Nitroxoline	6.25	6.25	1
106	Otilonium bromide	3.13	1.56	2
107	Penfluridol	>50	25	2
108	Piroctone olamine	50	50	1
109	Pitavastatin Calcium	1.56	0.78	2
110	Pramocaine hydrochloride	50	50	1
111	Tamoxifen Citrate	50	25	2
112	Teprenone	50	50	1
113	Vilazodone	>50	25	2
114	Visomitin	25	12.5	2
115	Abiraterone	>50	50	1
116	Armillarisin A	>50	>50	1
117	Atorvastatin hemicalcium salt	12.5	6.25	2
118	Auranofin	>50	50	1
119	Bepridil hydrochloride hydrate	50	50	1
120	Bithionol	50	50	1
121	Boceprevir	50	50	1
122	Bromperidol	25	25	1
123	Calcium lactate	25	25	1
124	Carmofur	>50	50	1
125	Cerivastatin sodium	1.56	1.56	1
126	Cetylpyridinium chloride monohydrate	6.25	3.13	2
127	Chloroxine	3.13	3.13	1
128	Ciclopirox olamine	>50	50	1
129	Degarelix	>50	50	1
130	Dichlorisone acetate	>50	50	1
131	Dronedarone	25	12.5	2
132	Eltrombopag	>50	50	1
133	Fingolimod hydrochloride	6.25	3.13	2
134	Flucytosine	>50	25	2
135	Fluspirilene	>50	50	1
136	Hexylresorcinol	50	50	1
137	Levamlodipine besylate	>50	>50	1
138	Nintedanib	>50	50	1
139	Octenidine dihydrochloride	1.56	1.56	1
140	Osimertinib mesylate	>50	25	2
141	Propofol	>50	50	1
142	Rosuvastatin calcium	25	25	1
143	Tamoxifen	12.5	12.5	1
144	Tavaborole	0.78	0.78	1
145	Terconazole	0.2	0.2	1
146	Ticagrelor	>50	25	2
147	Zinc Pyrithione	0.78	0.39	2

Annotation: NA: Not applicable. ■: 30 drugs are excluded as these drugs have potent antifungal activities. ■: 10 drugs were excluded as plumbagin antagonizes them. ■: 64 drugs were excluded as plumbagin slightly affected their antifungal activities.

**Table 4:** Drugs were evaluated for their interaction with plumbagin.

No.	Antifungal Agents	MIC (μM)		FIC	FICI	Outcome
		Alone	Combination			
1	HAL	50	1.56	0.03125	0.09375	Synergy
	Plumbagin	4	0.25	0.0625		
2	Ceritinib dihydrochloride	50	12.5	0.25	0.3125	Synergy
	Plumbagin	4	0.25	0.0625		
3	Vortioxetine hydrobromide	50	12.5	0.25	0.3125	Synergy
	Plumbagin	4	0.25	0.0625		
4	Disulfiram	25	3.13	0.125	0.375	Synergy
	Plumbagin	4	1	0.25		
5	Ponatinib	50	12.5	0.25	0.5	Synergy
	Plumbagin	4	1	0.25		
6	Tafenoquine succinate	25	6.25	0.25	0.5	Synergy
	Plumbagin	4	1	0.25		
7	Thonzonium bromide	12.5	3.13	0.25	0.5	Synergy
	Plumbagin	4	1	0.25		
8	Almonertinib hydrochloride	50	3.13	0.0625	0.5625	Addition
	Plumbagin	4	2	0.5		
9	Ebastine	25	3.13	0.125	0.625	Addition
	Plumbagin	4	2	0.5		
10	Menadione	50	6.25	0.125	0.625	Addition
	Plumbagin	4	2	0.5		
11	Sultiame	50	6.25	0.125	0.625	Addition
	Plumbagin	4	2	0.5		
12	Vilanterol trifenatate	50	6.25	0.125	0.625	Addition
	Plumbagin	4	2	0.5		
13	Amiodarone hydrochloride	50	12.5	0.25	0.75	Addition
	Plumbagin	4	2	0.5		
14	Cinacalcet	50	12.5	0.25	0.75	Addition
	Plumbagin	4	2	0.5		
15	Domiphen bromide	12.5	3.13	0.25	0.75	Addition
	Plumbagin	4	2	0.5		
16	Ilaprazole	50	12.5	0.25	0.75	Addition
	Plumbagin	4	2	0.5		
17	Rolapitant	50	12.5	0.25	0.75	Addition
	Plumbagin	4	2	0.5		
18	Amphotericin B	0.78	0.39	0.5	1	Addition
	Plumbagin	4	2	0.5		
19	Bleomycin hydrochloride	25	12.5	0.5	1	Addition
	Plumbagin	4	2	0.5		
20	Cetylpyridinium chloride	6.25	3.13	0.5	1	Addition
	Plumbagin	4	2	0.5		
21	Clioquinol	25	12.5	0.5	1	Addition
	Plumbagin	4	2	0.5		
22	Clomiphene (citrate)	25	12.5	0.5	1	Addition
	Plumbagin	4	2	0.5		
23	Fingolimod	6.25	3.13	0.5	1	Addition
	Plumbagin	4	2	0.5		
24	Pinaverium bromide	0.78	0.39	0.5	1	Addition
	Plumbagin	4	2	0.5		
25	Tegaserod (maleate)	50	25	0.5	1	Addition
	Plumbagin	4	2	0.5		
26	Telotristat ethyl	12.5	6.25	0.5	1	Addition
	Plumbagin	4	2	0.5		
27	Telotristat etiprate	12.5	6.25	0.5	1	Addition
	Plumbagin	4	2	0.5		
28	Triclosan	25	12.5	0.5	1	Addition
	Plumbagin	4	2	0.5		
29	Alectinib	50	50	1	2	Indifferent
	Plumbagin	4	4	1		
30	Benzethonium chloride	12.5	12.5	1	2	Indifferent
	Plumbagin	4	4	1		
31	Bosutinib	50	50	1	2	Indifferent
	Plumbagin	4	4	1		
32	Ceritinib	50	50	1	2	Indifferent
	Plumbagin	4	4	1		
33	Chlorhexidine	50	50	1	2	Indifferent
	Plumbagin	4	4	1		
34	Dacomitinib	50	50	1	2	Indifferent
	Plumbagin	4	4	1		
35	Ibudilast	50	50	1	2	Indifferent
	Plumbagin	4	4	1		
36	Magnolol	50	50	1	2	Indifferent
	Plumbagin	4	4	1		
37	Nilotinib monohydrochloride monohydrate	50	50	1	2	Indifferent
	Plumbagin	4	4	1		
38	Olmutinib	50	50	1	2	Indifferent
	Plumbagin	4	4	1		
39	Pimavanserin tartrate	50	50	1	2	Indifferent
	Plumbagin	4	4	1		
40	Sertindole	50	50	1	2	Indifferent
	Plumbagin	4	4	1		
41	Sonidegib	50	50	1	2	Indifferent
	Plumbagin	4	4	1		
42	Triflupromazine hydrochloride	50	50	1	2	Indifferent
	Plumbagin	4	4	1		
43	Vortioxetine	25	25	1	2	Indifferent
	Plumbagin	4	4	1		

Annotation: ■: 15 drugs are excluded because their relationship with plumbagin is indifferent. ■: 21 drugs were excluded because their relationship with plumbagin is additive.

**Table 5:** Drugs were evaluated for their interaction with menadione.

No.	Antifungal Agents	MIC (μM)		FIC	FICI	Outcome
		Alone	Combination			
1	HAL	50	1.56	0.03125	0.1563	Synergy
	Menadione	32	4	0.125		
2	Tafenoquine Succinate	25	12.5	0.5	0.625	Addition
	Menadione	32	4	0.125		
3	Ceritinib dihydrochloride	50	25	0.5	0.75	Addition
	Menadione	32	8	0.25		
4	Disulfiram	6.25	3.13	0.5	1	Addition
	Menadione	32	16	0.5		
5	Ponatinib	50	25	0.5	1	Addition
	Menadione	32	16	0.5		
6	Thonzonium bromide	12.5	6.25	0.5	1	Addition
	Menadione	32	16	0.5		
7	Vortioxetine hydrobromide	50	25	0.5	1	Addition
	Menadione	32	16	0.5		

**Table 6:** HAL combined with H_2_O_2_ against *C. albicans*.

Strain	Antifungal Agents	MIC		FIC	FICI	Outcome
		Alone	Combination			
SN152	HAL	>25 μM	0.78 μM	0.0156	0.2526	Synergy
	H_2_O_2_	3.13 mM	0.78 mM	0.25		
*hog1*Δ/Δ	HAL	1.56 μM	0.39 μM	0.25	0.5	Synergy
	H_2_O_2_	1.56 mM	0.39 mM	0.25		
*rad53*Δ/Δ	HAL	>25 μM	1.56 μM	0.03125	0.2813	Synergy
	H_2_O_2_	3.13 mM	0.78 mM	0.25		
*cap1*Δ/Δ	HAL	>25 μM	>25 μM	1	2	Indiferent
	H_2_O_2_	1.56 mM	1.56 mM	1		
*ybp1*Δ/Δ	HAL	>25 μM	>25 μM	1	2	Indiferent
	H_2_O_2_	0.78 mM	0.78 mM	1		
*gpx3*Δ/Δ	HAL	>25 μM	3.13 μM	0.0625	0.5625	addition
	H_2_O_2_	1.56 mM	0.78 mM	0.5		

**Table 7:** The interaction between HAL and NaCl.

Conditions	Agents	MIC		FIC	FICI	Outcome
		Alone	Combination			
in the absence of plumbagin	HAL	>25 μM	>25 μM	1	2	Indifferent
	NaCl	1000 mM	1000 mM	1		
in the presence of plumbagin (2 μg/mL)	HAL	0.78 μM	0.39 μM	0.5	1	Addition
	NaCl	62.5 mM	31.25 mM	0.5		

## Data Availability

Data are contained within the article.
